# An Assessment of Local Geometric Uncertainties in Polysilicon MEMS: A Genetic Algorithm and POD-Kriging Surrogate Modeling Approach

**DOI:** 10.3390/mi16020127

**Published:** 2025-01-23

**Authors:** Ananya Roy, Francesco Rizzini, Gabriele Gattere, Carlo Valzasina, Aldo Ghisi, Stefano Mariani

**Affiliations:** 1 Department of Civil and Environmental Engineering, Politecnico di Milano, Piazza Leonardo da Vinci 32, 20133 Milano, Italy; ananya.roy@polimi.it (A.R.); stefano.mariani@polimi.it (S.M.); 2 STMicroelectronics, Via Tolomeo 1, 20010 Cornaredo, Italy; francesco.rizzini@st.com (F.R.); gabriele.gattere@st.com (G.G.); carlo.valzasina@st.com (C.V.)

**Keywords:** micro electro-mechanical systems (MEMS), over-etching, uncertainty quantification, proper orthogonal decomposition (POD), kriging interpolation, genetic algorithm (GA)

## Abstract

On the way toward MEMS miniaturization, the quantification of geometric uncertainties stands as a primary challenge. In this paper, an approach that combines genetic algorithms and proper orthogonal decomposition with kriging surrogate modeling was proposed to accurately predict over-etch measures through an on-chip test device. Despite being fabricated on a single wafer under nominally identical manufacturing conditions, MEMS can display different responses under the same actuation, due to a different characteristic geometry. It is shown that the uncertainties, given in terms of over-etch values, were not only different from die to die but also within the same die, depending on the local geometric features of the device. Therefore, the proposed method provided an alternative solution to estimate the uncertainties in MEMS devices, relying only on the capacitance–voltage response. A statistical analysis was carried out based on a batch of devices tested in the laboratory. These tests and the estimation procedure allowed us to quantify the mean values of the over-etch relative to the target as +12.2 % at comb fingers, +10.0 % at the supporting springs, and −4.8 % at stoppers, showing noteworthy variability induced by the environment.

## 1. Introduction

The success of the miniaturization of micro-electromechanical systems (MEMS) [1] is based on the assumption that smaller and smaller devices can be designed and micro-fabricated, as their physical behavior is well known. The main belief of this reasoning is that the physics governing their behavior is simple, while manufacturing only proves difficult. However, by looking at the mechanical response of silicon-based inertial MEMS, this view is challenged whenever the uncertainties related to the material and to the geometrical features become of the same order as the expected response.

In [2], the tuning of the sensing frequencies of gyroscopes was set far from the drive by 10–15% due to the difficulty related to deep-reaction ion etching (DRIE) in releasing geometries within a prescribed tolerance on the beam width of 0.2 μm. In [3], a resonant T-beam was characterized in its nonlinear response, including the effects of epistemic and aleatory uncertainties, starting from geometrical discrepancies such as the lack of symmetry or length ratio not in accordance with the design and then moving on to mass and residual stress variations. Recently, in [4], it was shown that the process variations also affect the estimations of the coefficient of thermal expansion obtained with an in situ test structure featuring beams of small width.

Not only do the mean value drifts of device features matter, but also the variation of the relevant standard deviations. In fact, devices in the same batch or wafers can easily experience fluctuations in their parameters, affecting the working conditions. Several reasons are behind such a variation, ranging from the wafer bonding technology [5] to the local process conditions for the acid acting on the die during the DRIE and from the temperature of a certain manufacturing process step to the presence of impurities.

Whatever the reason, as stated in [6], “due to the process variations, no two microelectromechanical systems have the same geometry”. Geometric uncertainties refer to the deviations or variations in the dimensions and shapes of fabricated devices, away from their design specifications. The discrepancies arising due to micro-fabrication processes are here termed over-etch. The etching process in microsystem manufacturing can be claimed to be a critical technique leading to such etching defects. As etching is adopted to selectively remove material from the polycrystalline silicon (polysilicon) film, leading to microscale features and structures, it is essential in MEMS fabrication or semiconductors and other micro-fabricated devices [7]. In particular, in cases involving complex microstructures or different materials, etch rates can vary significantly across the substrate [8,9]: prolonged etching can result in the unintentional removal or damage of the underlying materials or structures that are meant to remain intact. This can compromise the mechanical stability and functionality of the microsystems or, at least, lead to scattering in their actual behavior.

From these premises, the need emerges to experimentally inspect the dimensions that are deemed critical. The most direct approaches, i.e., optical microscopy or scanning electron microscopy, allow for resolutions of 0.2 μm down to 0.01 μm in all the cases limited by uncertainties. Several alternative techniques were proposed to go beyond these limits: focusing on those feasible for controls during mass production, they can be based on experimental electromicrometrology [6,10], on the determination of resonance frequency [11], or on profilometry [12].

Uncertainties in the mechanical behavior of silicon can instead be related to the hypothesis of local material homogeneity. Within the present frame, they are related to the effects of heterogeneity due to the silicon grain morphology and orientation of the crystal lattices, see, e.g., [13,14], and to defects, e.g., sidewall grooves [15]. Regarding effective mechanical properties, when classical homogenization on representative volume elements cannot provide reliable or accurate results, see, e.g., [16], stochastic approaches raise interest [14,17]. In such cases, the aim is no more to obtain a single deterministic property but instead a probability distribution of it. As far as silicon strength is concerned, extensive experimental campaigns have been carried out over the years, recognizing the role of defects and making it possible to determine the relevant probability distribution for different manufacturing processes [18]. Although a Weibull distribution is claimed to be the most appropriate one, some issues remain open due to the unavoidable links between variations in the manufacturing process and strength itself [15].

Geometric uncertainties play a role in a number of microfabrication processes, but they do not necessarily deal with polysilicon as a structural material. For example, [19] described a procedure with more than 10 geometrical design parameters for a radio frequency MEMS made of electroplated gold thin film: a design of experiments for these parameters was used to generate an optimization problem, targeting the pull-in voltage as a specific quantity of interest. Alternatively, if the statistical distributions of the uncertain parameters affecting the output were known, such as in [20] for a nanocrystalline nickel thin film, a stochastic algorithm like Metropolis–Hastings Markov chain Monte Carlo could be used to find the best-fitting parameter values to maximize the likelihood with respect to the observed data. A good example related to the handling of geometrical and material uncertainties can be found in [21], where a radio frequency passive component made of electroplated gold was studied according to a response surface method, allowing us to reduce the number of numerical simulations in a multi-objective optimization problem.

For fluctuations in the geometry and in the material properties, the simplest approach adopted in the industry would be to neglect the underlying mechanisms and directly adjust the stiffness of the critical component by one of the methods available in the literature (for a review, see [22]), or by limiting the working conditions to avoid possible catastrophic events. In fact, uncertainties in the geometry do not only affect the MEMS operating conditions but also material characterization. Deterministic data reduction techniques need to be carefully and critically reviewed and sometimes integrated with uncertainty analysis, see, e.g., [23]. Following paths similar to those adopted at the macroscale, the variability of model parameters was treated by way of safety factors, resulting in an overly conservative design, then by Monte Carlo simulations, and finally, by designing a reliability scheme [24], or by robust optimization [25]. Most of these approaches were based on computationally expensive simulations, especially when high-fidelity finite element (FE) simulations were necessary. Alternative techniques were also explored, based on stochastic collocation methods [26], wherein the uncertainty is treated as a separate dimension in addition to the physical one, and a refinement of the polynomial chaos expansion was used in [4]. In [27], geometrical uncertainties such as over-etching and device (initial) position offset were instead identified using asymptotic homogenization, a numerical procedure used to recover the effective compliance of a MEMS filter affected by said uncertainties. It is worth mentioning that the procedure exploited information from C−V curves, thus allowing us to check the device’s behavior after all the microfabrication steps. In recent years, the concept of metamodels and model order reduction has helped us to address some of the issues related to the computational burden of uncertainty quantification [28], especially in the case of repeated simulations, to allow for the statistical effects of multiple uncertainty sources. By reducing the number of high-fidelity FE analyses within a reasonable number in the design space, a proxy of real-world solutions is set to attain accuracy and speed up the analysis. Recently, in [29], a supervised learning model based on artificial neural networks was used to surrogate the response of MEMS capacitive accelerometers: the high-fidelity FE analyses provided the results collected in a dataset, subsequently adopted to estimate the sensitivity of the accelerometer to the micro-fabrication uncertainties, namely mechanical offsets, over-etching, and film thickness.

In [30], an on-chip MEMS testing device was designed and tested to investigate the effects of microscale uncertainties on the performance and reliability of polysilicon-based devices. The challenge was to infer underlying uncertainties from standard capacitive measurements to avoid expensive experimental testing. In [31], a reduced-order analytical model of the MEMS device was coupled with a genetic algorithm (GA) to estimate the zero-voltage offset induced by asymmetries in over-etching, e.g., along compliant supporting springs connected to a proof mass. Unlike a previous work [32], where a particle filter was used within a Bayesian approach to estimate over-etching, a batch approach provided by the GA avoided instabilities near the pull-in. In [33], a stochastic method was used to estimate the effective Young’s modulus and the over-etching of the same devices by exploiting a reduced-order model obtained from high-fidelity FE analyses. Proper orthogonal decomposition (POD) was first used to get insights into the variation in the nonlinear response measured with C−V curves. Next, a kriging interpolation was adopted to inspect the variation in the dominant modes of the POD-based model within the parameter space. In this way, both mean values and standard deviations of the identified model parameters were obtained from a set of nominally identical devices.

In the current work, it is first shown that uniform geometry and size-independent etching-loss would not properly describe the experimental data, and a local estimation is provided for springs, comb fingers, and stoppers, actually representing the critical structural parts of the designed test structure. The testing device, already described in [34], is briefly recalled in Section 2 so that the effect due to a varying geometry on its stiffness and electro-mechanical response can be modeled. The MEMS testing device includes comb finger actuators, which have applications in various fields, including but not limited to electro-mechanical filters [35], optical shutters [36], microgrippers [37], voltmeters [38], inertial sensors for acceleration, and rotation sensing [39]. This study can provide useful insights for the MEMS community. Therefore, in Section 3 and Section 4, a numerical approach exploiting a GA-optimized POD-kriging model is presented to estimate geometric uncertainties, providing a faster and more accurate alternative to more conventional high-fidelity models of MEMS actuated by comb finger drives.

## 2. On-Chip Testing Device

**Figure 1:**
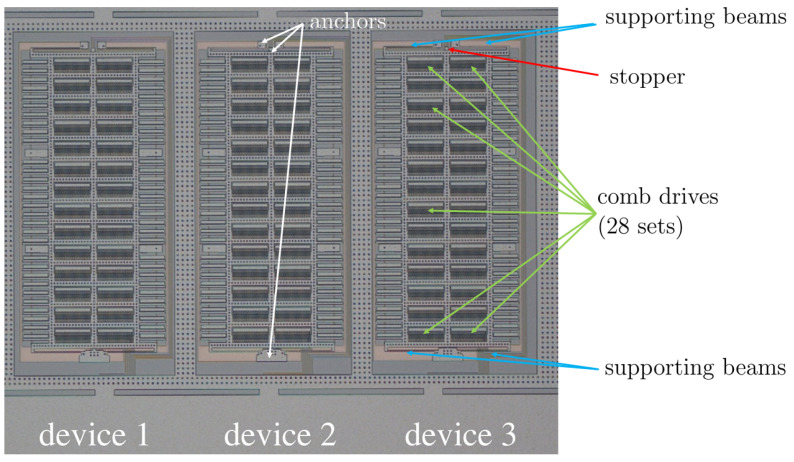
Micrograph of the considered MEMS testing devices.

To quantify the geometric uncertainties at the die level, in terms of over-etching and its possible variation due to the device geometry, a series of on-chip testing devices were designed ad hoc, see Figure 1. Each device consisted of a movable mass, termed a shuttle, supported by four beams anchored to the substrate. An electrostatic actuation was exploited to move the shuttle by way of comb drives and parallel plates. Twenty-eight stators in correspondence with the comb finger series were adopted to move the shuttle (upward in the reference frame of Figure 1) towards the stopper and to touch it. A further series of parallel plate capacitors was placed on the sides of the shuttle and designed to increase the electrostatic force so that the aforementioned stopper could be broken by further pushing the shuttle against it, see [34]. Since this latter capability of the device is not considered in the present work, aiming instead to assess geometrical uncertainties at the micro-scale rather than material strength-related ones, such stators are not considered hereafter.

MEMS devices were fabricated using the thick epitaxial layer for the micro-gyroscopes and accelerometers (ThELMA™) process developed by STMicroelectronics [40].

A single die housed three MEMS devices of this type, which share the same geometry of shuttle, suspension springs, and comb drives but feature stoppers of (slightly) different sizes, to also assess side effects related to the polysilicon strength. For a thorough discussion related to this issue, readers are referred to [34]. The three devices are therefore considered nominally the same.

To assure a reliable testing environment, a probe station with an optical microscope, micromanipulators, and an Agilent E4980A capacitance meter (Agilent Technologies, Santa Clara, CA, USA) was carefully positioned to interact seamlessly with the microsystems [34].

**Figure 2:**
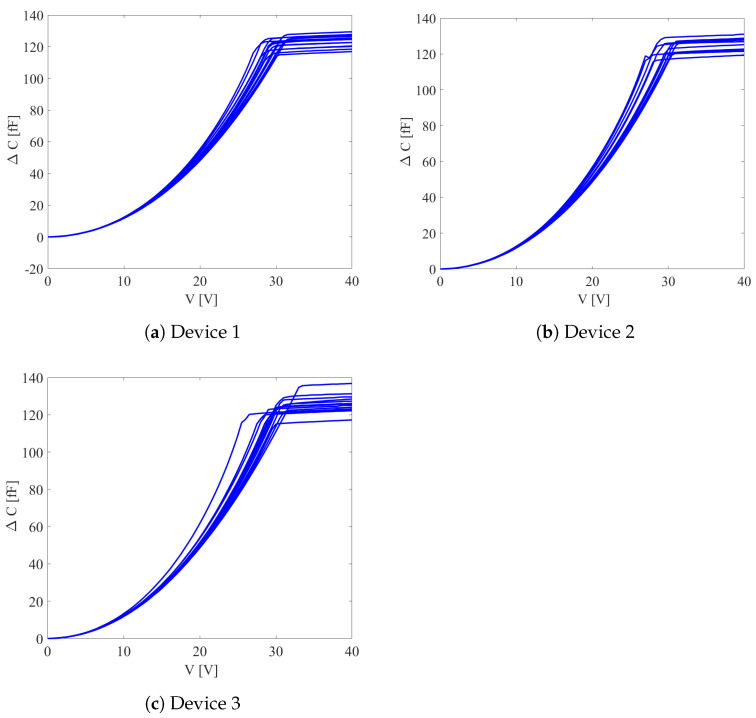
ΔC−V responses of the tested devices.

Under quasi-static loading conditions, a DC voltage bias *V* up to 40 V was applied smoothly and slowly to the shuttle, with the voltage incrementally raised by 0.5 V at each step while the stators remained grounded. Due to the applied DC bias voltage, the resulting electrostatic force moved the shuttle until it touched the stopper; next, since the shuttle could not move anymore, even larger values of the voltage bias did not lead to significant variations of the capacitance change ΔC measured at the comb fingers and used as sensor output to infer the response of the shuttle to the applied input. The ΔC−V curves relevant to all the tested devices are shown in Figure 2. A sequence of experimental tests was conducted on 12 dies to collect data and assess the scattering of over-etching. Although they were supposed to be all nominally the same, the plots show that curves differ both in the initial rising stage (when the shuttle moves) and in the subsequent plateau (after the contact between the shuttle and stopper) due to geometrical uncertainties linked to the etching phase of microfabrication.

In fact, as extensively discussed in [30,31], such spreading of the responses cannot be fully attributed to uncertainties related to the polysilicon morphology of the movable structure and must be properly estimated to foresee the response of real devices. Moreover, previous measurements of the residual stresses in the film excluded the relevance of this variable to affect the overall mechanical response, given the very low values. It is worth mentioning that, in general, residual stresses did affect the stiffness of suspension springs and, therefore, the ΔC−V response of the device.

In Figure 2, results are reported separately for three series of devices, which differ only in terms of the in-plane stopper size. As small variations in the target device geometry can lead to changes in the uncertainties of its actual geometry, results are kept independent in the analysis to follow. Compared to estimations of the inter-die and intra-die correlations between the statistics of the studied geometry-related uncertainties, over-etching is not assumed to be single-valued but constant throughout the die and, therefore, not affected by the surrounding environment. As it may vary depending on the location of the device component, a strategy is developed in Section 3 to allow for different values or statistical distributions at combs, at suspension springs, and at the place of contact between the shuttle and stoppers. Due to the limited information available from the ΔC−V curve, only three properties were estimated and considered representative of the entire device.

## 3. Methodology: An Integrated POD-Kriging and Inverse GA Problem Solver

To provide an interpretation of the results of the experimental campaign specifically dealing with local uncertainty linked to over-etching, numerical simulations were carried out with the FE software COMSOL 6.3 Multiphysics™. In the analysis, each FE simulation featured different (single-value) homogeneous over-etching at the supporting beams and in comb drive areas. In this way, a grid of solutions was built in the space of the design variables, which will be processed next accordingly.

**Figure 3:**
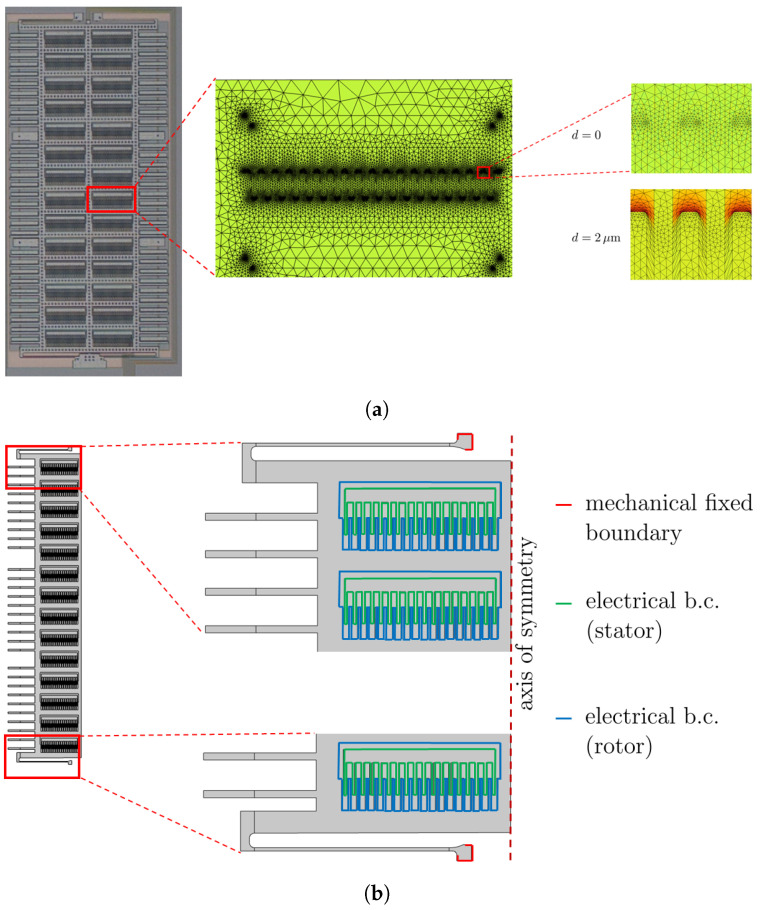
(**a**) Re-meshing in the air domain due to shuttle motion and (**b**) boundary conditions adopted in the finite element high-fidelity model (exploiting problem symmetry).

The numerical results were then compared with the experimental data, and an inverse problem was solved to match, at best, the measured responses. The high-fidelity FE model of one half of the device, in view of the problem symmetry, see Figure 3, allowed for electro-mechanical coupling and electrical domain re-meshing to accommodate large shuttle displacements during actuation (Figure 3a). It is, in fact, rather well known, see [41], that even small displacements structure-wise, which allow keeping a linearized kinematics to describe the motion, can be large enough fluid-wise in the case of small gaps between the rotor and stator so that an update of the configuration and, therefore, of the mesh becomes necessary. The mesh consisted of linear triangular continuum elements for the solid domain (stators and shuttle) and linear triangular elements for the electrical domain in between the comb fingers. To reduce the computational burden, no electrical domains were considered out of the solid framework. The nodes in the electrical domain have only potential degrees of freedom to describe the varying electric field during the shuttle motion. Electrical boundary conditions were applied to the solid edges facing the fluid between the comb fingers, see Figure 3b: the shuttle was grounded, and an increasing DC voltage, from zero up to 40 V, was applied to all the stators. The capacitance change was computed globally between the stators on one side and all the surrounding surfaces of the interdigitated comb fingers, the surfaces facing the back of the stators, and the lateral surfaces of the rotor on the other side. Finally, mechanical boundary conditions were adopted at the anchors and at the stators to prevent rigid body motions; see Figure 3b.

In what follows, the three procedural steps adopted to reduce the computational burden of the inverse problem and of its solution are briefly described. A surrogate of the high-fidelity FE model is thereby set and adopted within an iterative procedure based on a GA.

### 3.1. Proper Orthogonal Decomposition

As generated FE models are computationally expensive, POD was adopted to dig into solutions and look for relevant patterns in them. In general terms, model-order reduction techniques aim to provide a reduced-order model that retains the essential features of a complex, higher-dimensional one, e.g., by projecting the solution of the numerical analysis onto a subspace of reduced dimensionality [42]. This is achieved by identifying a set of basis vectors in the projection subspace. Among various strategies for selecting these bases, POD uses so-called snapshots that capture the system’s response under specific inputs [43].

Based on the simulation results, a snapshot matrix S is assembled to collect ΔC−V curves at different over-etching values. In concrete terms, S reads as follows:

(1) S={ΔC(1),ΔC(2),…,ΔC(N)},

where $\Delta C\left(i\right)\in R^{M}$ is the *i*-th computed set of ΔC values at an increasing applied voltage *V*; *M* is the number of points in each ΔC−V curve, here assumed constant for all the simulations; *N* is the total number of combinations of the input parameters (i.e., over-etches) adopted to build the snapshot matrix itself.

Next, a singular value decomposition is performed on the snapshot matrix, leading to the following:

(2) $$S=U\Sigma V^{T},$$

where $U=\left[\Phi_{1},\Phi_{2},\ldots ,\Phi_{M}\right]\in R^{M\times M}$ and $V^{T}=\left[\xi_{1},\xi_{2},\ldots ,\xi_{N}\right]\in R^{N\times N}$ are left and right singular vectors of S, respectively; $\Sigma \in R^{M\times N}$ is the matrix gathering (non-negative) singular values $\sigma_{i}$ of *S*, which are sorted in descending order as $\sigma_{1}⩾\sigma_{2}⩾\cdots ⩾\sigma_{M}$. The first *r* leading singular vectors in U are used to deliver an optimal reduced basis space, according to the following:

(3) $$\Phi_{POD}=\mathrm{span}\left\{\Phi_{1},\Phi_{2},\ldots ,\Phi_{r}\right\}\in R^{M\times r},$$

and the entire solution can be approximated with $\Delta C\approx \Phi_{POD}\;\Delta C_{r}$, projecting the solution to a lower dimensional subspace spanned by $\Phi_{POD}$. The reduced-order model is given by the retained degrees of freedom ${\Delta C}_{r}\in R^{r}$, with r≪M. The dimension *r* of the POD is set by evaluating the relative importance of the singular values so as to achieve the following:

(4) $$\frac{\sum_{i=1}^{r}\sigma_{i}^{2}}{\sum_{i=1}^{M}\sigma_{i}^{2}}\geq 1-\epsilon ,$$

where ϵ is adopted small enough to catch, at best, the full-order solution (usually $10^{-2}÷$ $10^{-3}$).

### 3.2. Kriging Interpolation

Since the POD procedure described in what precedes can provide a reduced-order model for the input parameters adopted in the original analyses to assemble the snapshot matrix, an additional procedure is necessary to describe how the reduced-order model changes with the input parameters in a hopefully smooth manner.

Kriging interpolation is used here to surrogate the numerical model at any intermediate parameter value based on the observations at sampled locations considered by the POD step [44,45]. The core idea behind kriging is to compute the value of a random field at an unsampled point by a weighted sum of observed values, with weights optimally chosen based on the spatial correlation among data points. Although the goal of the present study is the assessment of statistical properties of over-etching and the effects of the etch environment on it, it must be kept in mind that such statistics are not directly considered in kriging; similarly, using the kriging interpolation on random fields provides the (additional and necessary) means to estimate them.

The device response, as obtained with the kriging interpolation, can thus be given as follows:

(5) $$\Delta C_{r}^{*}\left(p_{0}\right)=\sum_{i=1}^{n_{s}}\lambda_{i}\;\Delta C_{r}\left(p_{i}\right),$$

where $\Delta C_{r}^{*}\left(p_{0}\right)$ is the estimate at the unsampled location $p_{0}$; $\Delta C_{r}\left(p_{i}\right)$ is the observed solution at the *i*-th sampled location $p_{i}$, i.e., at the chosen over-etch parameter values at the suspension springs and at comb fingers, see Section 4; $\lambda_{i}$ is the *i*-th relevant weight; $n_{s}$ is the number of sampled locations used for the interpolation. The weights $\lambda_{i}$, $i=1,\ldots ,n_{s}$ are determined so that the estimator is unbiased and the variance of the estimation error is minimized. These two considerations lead to a system of equations known as the kriging system, see [44]:

(6) $$\begin{array}{cc} \sum_{j=1}^{n_{s}} & \lambda_{j}\;\gamma \left(p_{i},p_{j}\right)+\mu =\gamma \left(p_{i},p_{0}\right),\\ \sum_{j=1}^{n_{s}} & \lambda_{j}=1, \end{array}$$

where $\gamma (p_{i},p_{j})$ is the semivariogram value between locations $p_{i}$ and $p_{j}$; $\gamma (p_{i},p_{0})$ is the semivariogram value between the sampled location $p_{i}$ and the unsampled location $p_{0}$; μ is a Lagrange multiplier introduced to ensure the unbiasedness of the estimator.

The semivariogram γ(h), which is a key component in kriging, is defined as half the variance of the difference between field values at two different locations separated by a lag distance h:

(7) $$\gamma \left(h\right)=\frac{1}{2}\;\mathrm{Var}\left[Z\left(p\right)-Z\left(p+h\right)\right].$$

Thus, the semivariogram characterizes the spatial autocorrelation of the data and is typically modeled based on observed data. Once the semivariogram model is established and the kriging system is solved in terms of weights $\lambda_{i}$, the solution at an unsampled location can be predicted. Therefore, kriging results is advantageous compared to alternate methods, as it provides both estimates of the variable of interest at unsampled locations and of the uncertainty (variance) of such predictions.

### 3.3. Genetic Algorithm

To foresee the value of the over-etch, a GA is used to handle the outcome of the POD-kriging model. As known and detailed in the following, a GA can be exploited as an iterative solver of inverse problems, thereby requiring sufficient computational resources. This is the reason why the surrogate model built upon POD and kriging is necessary to efficiently obtain the sought device response and location-dependent estimates of over-etching.

The GA is a meta-heuristic approach inspired by the process of natural selection and genetics [46]. To seek solutions to optimization problems, GAs operate on a population of potential candidates using the principle of survival of the fittest to iteratively lead to better approximations. At each step, the algorithm selects individuals from the current population to be parents and uses them to produce children for the next generation. Over successive generations, the population is expected to evolve toward an optimal solution. The basic steps of a GA are as follows:

- Initialization: an initial population of *n* individuals is randomly generated. Each individual, also known as a chromosome, is a potential solution to the problem and is typically represented as a string of characters or numbers.
- Fitness evaluation: The fitness of each individual in the population is evaluated. The fitness function quantitatively measures how close a given solution is to the optimum and is here assumed as the mean square error between the surrogate response and the experimental data to match.
- Selection: Pairs of individuals (parents) are selected for reproduction based on their fitness scores. Better-fit individuals have a higher chance of being selected, and this can be represented by a probability proportional to fitness.
- Crossover (recombination): With a crossover probability, pairs of parents are combined to produce offspring for the next generation. Crossover is used to vary the programming of chromosomes from one generation to the next.
- Mutation: With a mutation probability, small random changes are made in offspring individuals to maintain genetic diversity within the population.
- Replacement: A new generation is formed by selecting individuals from the current generation and new offspring based on their fitness values.

The algorithm stops when either a maximum number of generations is produced or if a satisfactory fitness level is reached by the current population. The GA stands as a versatile algorithm, although the choice of representation, fitness function, selection mechanism, and other parameters, e.g., population size and mutation rate, can significantly affect its performance.

## 4. Results

**Figure 4:**
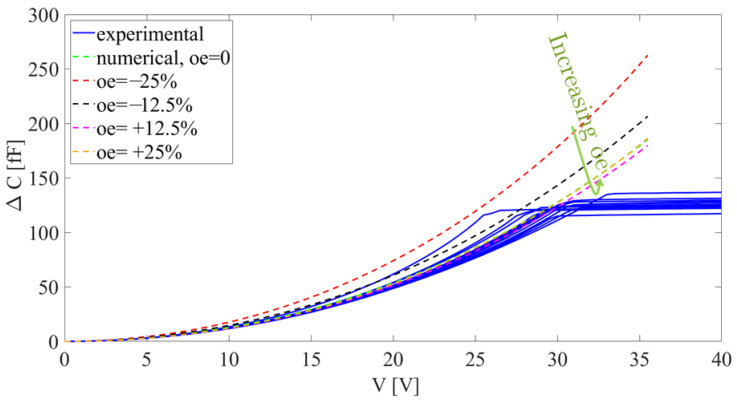
Exemplary comparison between the experimental data (blue lines) and the full-order model results (dotted lines) in case a uniform over-etching distribution is assumed in the analyses.

The inverse problem is here set by considering two parameters related to over-etching interpreted hereafter as the difference between a desired target and the actual value after micro-fabrication. The difference will be represented as a percentage of a possible discrepancy equal to 0.6 μm (±0.3 μm). Both over-etching at the comb fingers, denoted by $O_{c}$, and over-etching of the beams, denoted by $O_{b}$, were considered. In fact, by allowing for a single parameter like in [30] to homogeneously describe over-etching throughout the entire die, the model produced curves that, starting from the top, move towards the experimental blue curves in Figure 4; however, when a relative over-etching value of about +12.5% was reached, the red dashed curves rose again, and the distance between the numerical and experimental curves increased. Since such solutions did not allow us to match the experimental data collected by testing different devices with a reasonable accuracy, the strategy characterized by two over-etching values at the two locations handled simultaneously was explored next.

**Figure 5:**
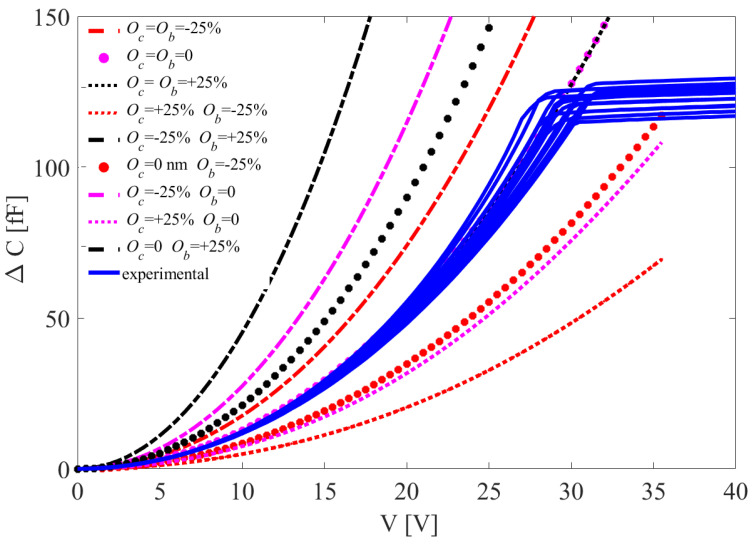
Comparison between full-order numerical model outcomes and experimental data (device 1).

The parametric space defined by $O_{c}$ and $O_{b}$ was inspected within the range $-25\%\leq O_{b},O_{c}\leq +25\%$. Full-order, high-fidelity FE models of the MEMS devices were adopted to compute the device responses for only nine combinations of $O_{c}$ and $O_{b}$ reported in the legend of Figure 5.

The numerical curves cover the part of the ΔC−V plane that fits the experimental ones, thereby allowing the kriging interpolation to represent the best-fitting one for each experiment.

To reduce the computational complexity, according to the procedure described in Section 3.3, POD was adopted. The kriging interpolation method was next used to surrogate the models for any intermediate values of $O_{c}$ and $O_{b}$ within the parameter space. The objective of GA optimization is to reduce the error between responses of the numerical model and the experimental curves. The GA was exploited by considering a population size of 100 individuals, a crossover probability of 85%, and a mutation probability of 15%.

**Figure 6:**
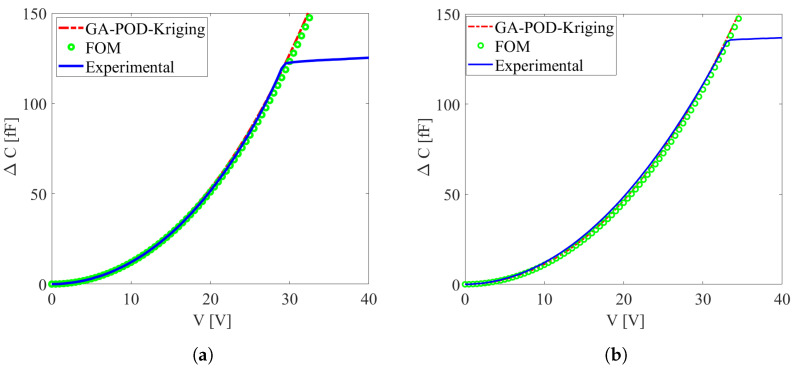
Comparison between the experimental, reduced-order, and full-order model ΔC−V curves for (**a**) device 1 and (**b**) device 3 taken from an exemplary die.

The values of $O_{c}$ and $O_{b}$ for each testing device were then estimated, and Figure 6a presents the responses of device 2 in an exemplary die, incorporating the optimized over-etch values of $O_{c}=O_{b}$ = +13.8%. Similarly, Figure 6b presents the responses of device 3 in the same exemplary die, featuring estimated over-etching values of $O_{c}$ = +6.5% and $O_{b}$ = +1.3%. In the charts, the solid blue lines represent the experimental data, the green lines depict the response of the full-order model for specific over-etching conditions, and the red lines stand for the response derived from the GA-based POD-kriging method. It must be noted that the full-order model solution is reported in these plots only to testify to the accuracy (evaluated ex-post) of the adopted reduced-order modeling strategy handled in the solution of the inverse problem, while it did not enter into any calculations during the analysis. Thus, it resulted that, for the same die, constant over-etching was not always useful in matching the experimental data: in the exemplary die, it worked for device 1 but not for device 3.

**Figure 7:**
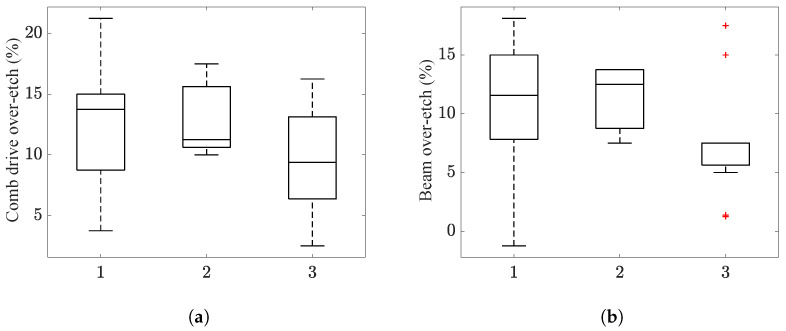
Box plots representing the scattering of the estimated values of over-etches (**a**) $O_{c}$ and (**b**) $O_{b}$.

By way of the GA-POD-kriging methodology, the over-etching values for all devices across the entire die array were computed, and they are, respectively, reported in Figure 7a,b in terms of box plots of the estimated $O_{c}$ and $O_{b}$ values. Device 1 and device 2 showed comparable $O_{c}$ and $O_{b}$ scattering, with a mean value of around +12.5%, while device 3 showed smaller mean values, on the order of +7.5%. The most scattered results are related to device 1, probably due to its position in the die depicted in Figure 1. More than that, outcomes relevant to device 3 could be affected by a nearby structure, which is not discussed in this work.

**Figure 8:**
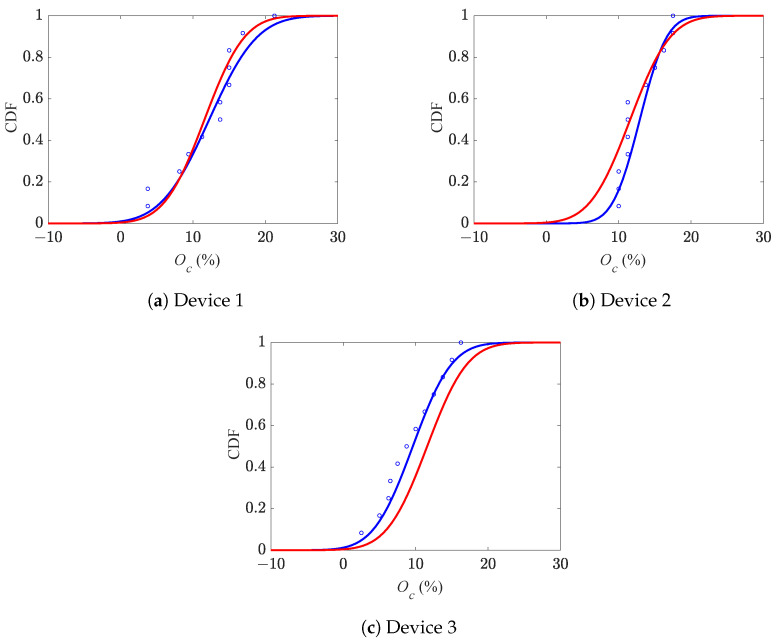
Cumulative distribution functions of comb drive over-etching $O_{c}$.

The relevant cumulative distribution functions (CDFs) of $O_{c}$ are displayed in Figure 8 for the three series of devices, as obtained by employing a normal distribution to fit all the results: the blue curves are related to the best fitting of the results relevant to each single batch, while the red ones are related to all the devices. It turned out that the mean values of $O_{c}$ resulted in +13.6% for device 1, +12.8% for device 2, and +9.5% for device 3; by considering all the devices, the overall mean value resulted instead in +12.1%. The values of the standard deviation of $O_{c}$ were 5.2%, 3.0%, and 4.2% for devices 1, 2, and 3, respectively, and 4.5% for all the devices. The plots show that the CDFs have different slopes due to the different values of the aforementioned standard deviations. In some cases (devices 1 and 3), the blue and red curves run parallel, in others (device 2), they cross each other due to the different mean values, too. Since the red CDF is reported as a reference for all the solutions, it is clear that even moving inside the same die, the estimated value of $O_{c}$ changes and, therefore, in a cumulative manner, also its probabilistic features. More specifically, as already reported in Figure 7, device 3 is characterized by responses that are rather dissimilar from the others.

**Figure 9:**
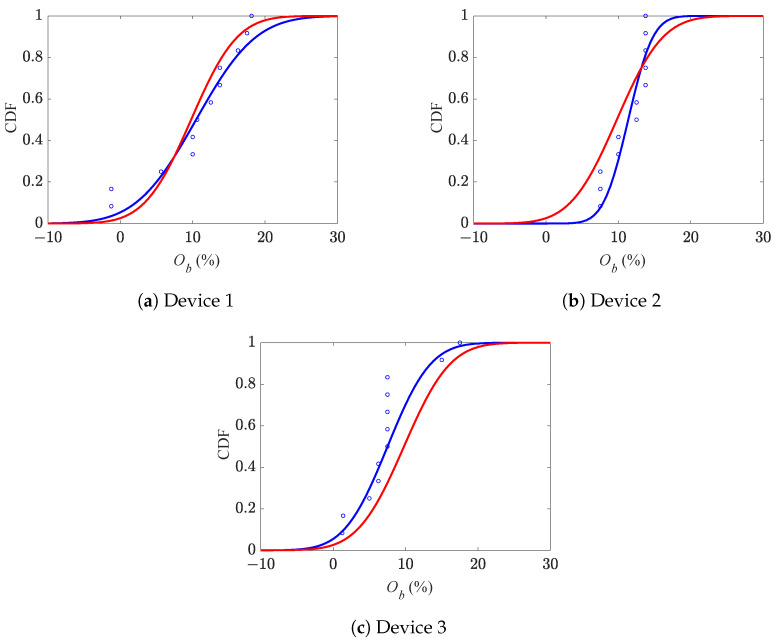
Cumulative distribution functions of beam over-etching $O_{b}$.

In a similar way, the CDFs of $O_{b}$ are shown in Figure 9. For this over-etching feature, the mean values were obtained as +11.5%, +11.5%, and +7.0% for devices 1, 2, and 3, respectively, and +10.0% for all devices. The relevant standard deviations were instead 5.8%, 2.8%, and 4.8% for devices 1, 2, and 3, respectively, and 5.2% for all devices. The comparison between the two CDFs in these plots shows again that device 3 was characterized by a rather different response if compared to the other ones.

**Figure 10:**
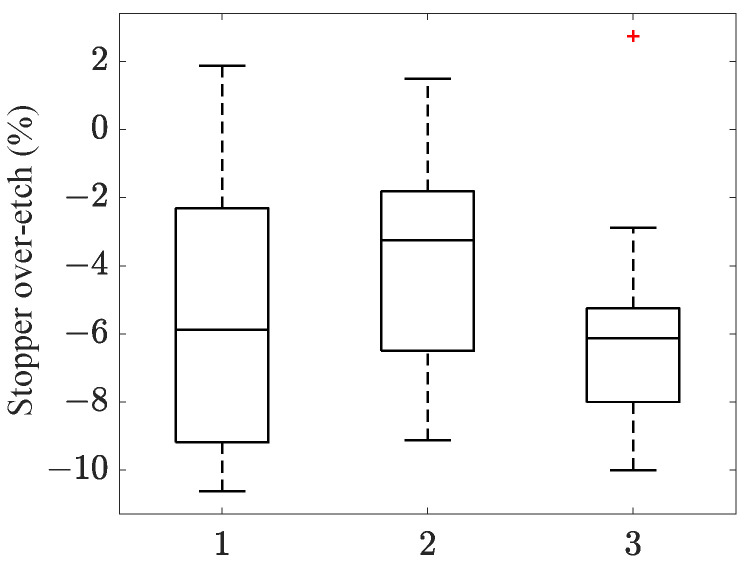
Box plots representing the scattering of estimated values of over-etching $O_{s}$.

To obtain the estimate of over-etching $O_{s}$ at the stopper, the shuttle displacement *d* corresponding to the plateau in the ΔC−V curve, as seen in Figure 2, was also computed. Moving from the high-fidelity model results linked to the corresponding estimated $O_{c}$ and $O_{b}$ values and by way of kriging interpolation, the statistics of *d* were obtained. The gap between the shuttle and the stopper was set to 0.8 μm at the design stage. Figure 10 provides relevant scattered estimates for all the devices.

**Figure 11:**
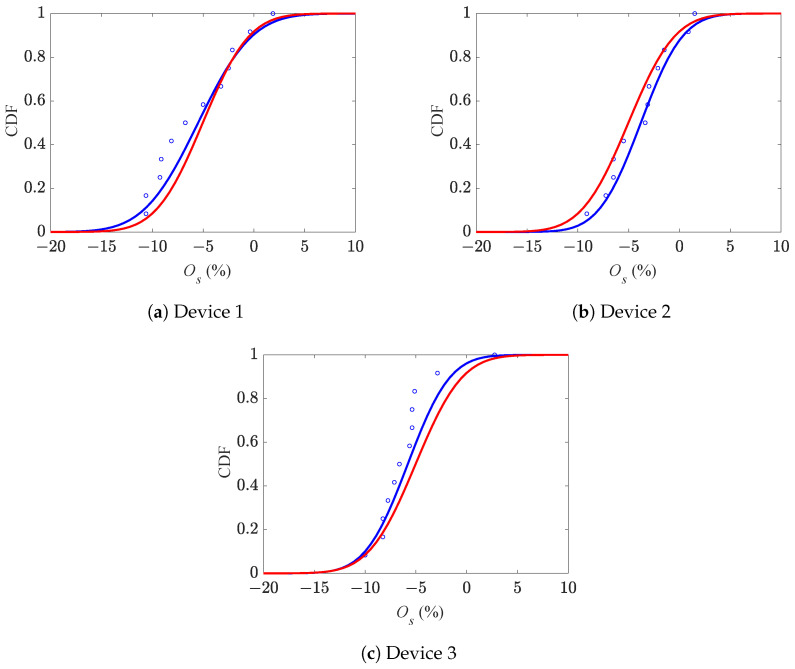
Cumulative distribution functions of stopper over-etching $O_{s}$.

The CDFs of $O_{s}$ of the three batches of devices were again fitted employing normal distributions and are depicted in Figure 11. The relevant mean values about the target (assumed uniform at the die level) were identified as −5.0%, −3.8%, and −5.5% for devices 1, 2, and 3, respectively, and −4.8% for all the devices. The reported negative values mean the etch stage was less effective than expected at the stopper position, as all the numbers should be considered relative to the already mentioned target. The corresponding standard deviations instead read 3.8%, 3.3%, and 3.3% for devices 1, 2, and 3, and 3.5% for all the devices.

The estimated overall mean value of $O_{s}$ was around half those relevant to $O_{b}$ and $O_{c}$. While it is expected to observe a smaller value of over-etching where the gap between the facing surfaces of the rotor and stator is small, the present results testified that an accurate estimation of its magnitude and of its dependence on the device features must be conducted on the basis of a full geometry-driven analysis.

**Figure 12:**
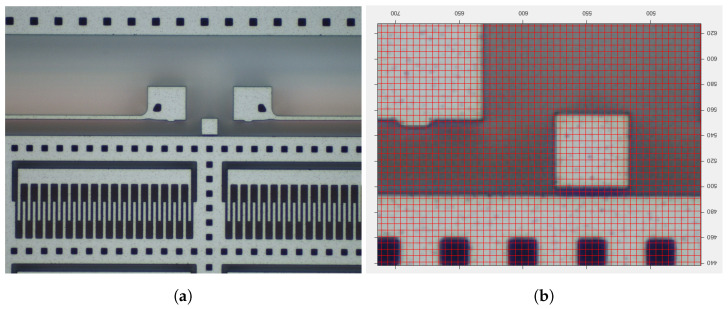
(**a**) Magnified view of the stopper area, and (**b**) close-up at the gap between the shuttle and the stopper.

To validate the obtained results, the gap between the shuttle and the stopper was measured through optical means on some tested devices, yielding a reference value of 1.120 μm; see Figure 12. Over-etching at the stopper thus turned out to be approximately −5%, which is in fairly good agreement with the output of the GA POD-kriging approach.

## 5. Conclusions

In this paper, a methodology was offered to estimate non-uniform geometric uncertainties at the microscale through on-chip testing devices. The procedure was based on a combination of proper orthogonal decomposition and kriging surrogate modeling to deliver a reduced-order, efficient solution to compute the response of the devices to a driving signal. A genetic algorithm was next adopted to estimate the scattering of over-etching at the flexes of the movable structure and at the comb fingers, used for actuation and read-out.

A set of devices, all supposed to be nominally identical, were experimentally tested to obtain the input–output characteristics in terms of ΔC−V curves to be matched by the numerical surrogate model. At variance with former activities, for the present devices, it was shown that a uniform, position-insensitive estimation of over-etching did not allow the matching of experimental data.

Results obtained with the genetic algorithm showed that, even if the devices were taken from the same die, they display different over-etching values at different locations (beam versus comb finger drive areas) that, in the end, affect their response to the external actions. Normal distributions were finally adopted to fit the over-etching scattered data at the two aforementioned locations and at stoppers, leading to estimated mean values away from the target equal to +12.2% at the comb fingers, +10.0% at the supporting beams, and −4.8% and at the stoppers, respectively, and standard deviations on the order of 3÷5%.

Therefore, the proposed approach offered an accurate and efficient alternative solution to traditional optical methods to estimate geometric uncertainties of micro-scale devices, with significant potential benefits for the MEMS industry. In particular, the quantification of the uncertainty in the neighborhood of stoppers could be helpful to better characterize the polysilicon strength studied in [34], also accounting for the fluctuations in the local MEMS geometry. In future activities, the geometry- and material-dependent uncertainties are going to be accounted for simultaneously in a truly statistical model of the overall behavior of the MEMS testing device.

## Data Availability

The data presented in this study are available on request from the corresponding author. The data are not publicly available due to industrial requirements.

## References

[1] Gad-el Hak M. (2001). The MEMS Handbook.

[2] Weinberg M.S., Kourepenis A. (2006). Error sources in in-plane silicon tuning-fork MEMS gyroscopes. J. Microelectromech. Syst..

[3] Goyal R., Bajaj A.K. (2018). Uncertainty quantification in a resonant nonlinear MEMS structure. Int. J. Non-Linear Mech..

[4] Zhao L.F., Zhou Z.F., Huang Q.A. (2024). Uncertainty quantification with high-dimensional correlated process variations for an in-situ thermal expansion coefficient test structure. Sens. Actuators A Phys..

[5] Chen H.L., Chiang K.N. The Effect of Geometric and Material Uncertainty on Debonding Warpage in Fan-Out Panel Level Packaging. Proceedings of the 2023 24th International Conference on Thermal, Mechanical and Multi-Physics Simulation and Experiments in Microelectronics and Microsystems (EuroSimE).

[6] Li F., Clark J.V. (2012). Self-Calibration for MEMS with Comb Drives: Measurement of Gap. J. Microelectromech. Syst..

[7] Kolasinski K.W. (2008). Chapter 16 Growth and Etching of Semiconductors. Handb. Surf. Sci..

[8] Williams K.R., Muller R.S. (1996). Etch rates for micromachining processing. J. Microelectromech. Syst..

[9] Williams K.R., Gupta K., Wasilik M. (2003). Etch rates for micromachining processing-Part II. J. Microelectromech. Syst..

[10] Li F., Peroulis D., Clark J.V. (2014). Measuring Effective Flexure Width by Measuring Comb Drive Capacitance. J. Microelectromech. Syst..

[11] Gupta R.K. (2000). Electronically probed measurements of MEMS geometries. J. Microelectromech. Syst..

[12] Conroy M., Armstrong J. (2005). A comparison of surface metrology techniques. J. Phys. Conf. Ser..

[13] Ballarini R., Mullen R., Heuer A. (1999). The effects of heterogeneity and anisotropy on the size effect in cracked polycrystalline films. Fracture Scaling.

[14] Ghisi A., Mariani S. (2019). Effect of imperfections due to material heterogeneity on the offset of polysilicon MEMS structures. Sensors.

[15] DelRio F.W., Cook R.F., Boyce B.L. (2015). Fracture strength of micro- and nano-scale silicon components. Appl. Phys. Rev..

[16] Cho S., Chasiotis I. (2007). Elastic properties and representative volume element of polycrystalline silicon for MEMS. Exp. Mech..

[17] Ostoja-Starzewski M. (2006). Material spatial randomness: From statistical to representative volume element. Probabilistic Eng. Mech..

[18] Bernal R.A. (2021). On the application of Weibull statistics for describing strength of micro and nanostructures. Mech. Mater..

[19] Saleem M.M., Somà A. (2015). Design optimization of RF-MEMS switch considering thermally induced residual stress and process uncertainties. Microelectron. Reliab..

[20] Kolis P., Bajaj A.K., Koslowski M. (2017). Quantification of Uncertainty in Creep Failure of RF-MEMS Switches. J. Microelectromech. Syst..

[21] Iannacci J., Tagliapietra G., Bucciarelli A. (2022). Exploitation of response surface method for the optimization of RF-MEMS reconfigurable devices in view of future beyond-5G, 6G and super-IoT applications. Sci. Rep..

[22] de Laat M., Pérez Garza H., Herder J., Ghatkesar M. (2016). A review on in situ stiffness adjustment methods in MEMS. J. Micromech. Microeng..

[23] Muntwyler S., Kratochvil B.E., Beyeler F., Nelson B.J. (2010). Monolithically Integrated Two-Axis Microtensile Tester for the MechanicalCharacterization of Microscopic Samples. J. Microelectromech. Syst..

[24] Reh S., Lethbridge P., Ostergaard D. Quality based design and design for reliability of micro electro mechanical systems (MEMS) using probabilistic methods. Proceedings of the 2000 International Conference on Modeling and Simulation of Microsystems.

[25] Han J.S., Kwak B.M. (2001). Robust optimal design of a vibratory microgyroscope considering fabrication errors. J. Micromech. Microeng..

[26] Agarwal N., Aluru N. (2009). Stochastic analysis of electrostatic MEMS subjected to parameter variations. J. Microelectromech. Syst..

[27] Faraci D., Zega V., Nastro A., Comi C. (2022). Identification of MEMS Geometric Uncertainties through Homogenization. Micro.

[28] Bi S., Beer M., Cogan S., Mottershead J. (2023). Stochastic Model Updating with Uncertainty Quantification: An Overview and Tutorial. Mech. Syst. Signal Process..

[29] Zacchei F., Rizzini F., Gattere G., Frangi A., Manzoni A. (2024). Neural networks based surrogate modeling for efficient uncertainty quantification and calibration of MEMS accelerometers. Int. J. Non-Linear Mech..

[30] Mirzazadeh R., Ghisi A., Mariani S. (2018). Statistical Investigation of the Mechanical and Geometrical Properties of Polysilicon Films through On-Chip Tests. Micromachines.

[31] Mirzazadeh R., Mariani S. (2017). Uncertainty quantification of microstructure-governed properties of polysilicon MEMS. Micromachines.

[32] Mirzazadeh R., Eftekhar Azam S., Mariani S. (2016). Micromechanical Characterization of Polysilicon Films through On-Chip Tests. Sensors.

[33] Mirzazadeh R., Mariani S. (2018). Mechanical characterization of polysilicon MEMS: A hybrid TMCMC/POD-kriging approach. Sensors.

[34] Vicentini Ferreira do Valle T., Mariani S., Ghisi A., De Masi B., Rizzini F., Gattere G., Valzasina C. (2023). MEMS Reliability: On-Chip Testing for the Characterization of the Out-of-Plane Polysilicon Strength. Micromachines.

[35] Lin L., Howe R., Pisano A. (1998). Microelectromechanical filters for signal processing. J. Microelectromech. Syst..

[36] Jaecklin V., Linder C., de Rooij N., Moret J.M., Vuilleumier R. Optical microshutters and torsional micromirrors for light modulator arrays. Proceedings of the [1993] Proceedings IEEE Micro Electro Mechanical Systems.

[37] Kim C.J., Pisano A., Muller R. (1992). Silicon-processed overhanging microgripper. J. Microelectromech. Syst..

[38] Legtenberg R., Groeneveld A.W., Elwenspoek M. (1996). Comb-drive actuators for large displacements. J. Micromech. Microeng..

[39] Crescenzi R., Castellito G.V., Quaranta S., Balucani M. (2020). Design of a Tri-Axial Surface Micromachined MEMS Vibrating Gyroscope. Sensors.

[40] Corigliano A., De Masi B., Frangi A., Comi C., Villa A., Marchi M. (2004). Mechanical characterization of polysilicon through on-chip tensile tests. J. Microelectromech. Syst..

[41] Corigliano A., Dossi M., Mariani S. (2013). Domain decomposition and model order reduction methods applied to the simulation of multi-physics problems in MEMS. Comput. Struct..

[42] Schilders W.H., der Vorst H.A.V., Rommes J. (2008). Model Order Reduction: Theory, Research Aspects and Applications.

[43] Kramer B., Willcox K.E. (2019). Nonlinear Model Order Reduction via Lifting Transformations and Proper Orthogonal Decomposition. AIAA J..

[44] van Beers W., Kleijnen J. Kriging interpolation in simulation: A survey. Proceedings of the 2004 Winter Simulation Conference.

[45] Chica-Olmo M., Luque-Espinar J.A. (2002). Applications of the local estimation of the probability distribution function in environmental sciences by kriging methods. Inverse Probl..

[46] Lambora A., Gupta K., Chopra K. Genetic Algorithm—A Literature Review. Proceedings of the 2019 International Conference on Machine Learning, Big Data, Cloud and Parallel Computing (COMITCon).

